# The epigenetic downregulation of LncGHRLOS mediated by RNA m6A methylase ZCCHC4 promotes colorectal cancer tumorigenesis

**DOI:** 10.1186/s13046-024-02965-5

**Published:** 2024-02-07

**Authors:** Ke Chen, Jingcheng Zhang, Lei Meng, Lingshang Kong, Ming Lu, Zhengguang Wang, Wenbin Wang

**Affiliations:** 1 Vascular Surgery Department, Nanjing Drum Tower Hospital Affiliated to Medical School of Nanjing University, Nanjing, China; 2 Department of Surgery, Klinikum rechts der Isar, School of Medicine, Technical University of Munich, Munich, Germany; 3 General Surgery Department, The Second Affiliated Hospital of Anhui Medical University, Hefei, China; 4 General Surgery Department, Anhui Provincial Hospital, Hefei, China; 5 General Surgery Department, The First Affiliated Hospital of Anhui Medical University, Hefei, China

**Keywords:** ZCCHC4, LncRNAGHRLOS, Colorectal cancer, N6-methyladenosine, KDM5D

## Abstract

**Background:**

m6A modification is currently recognized as a major driver of RNA function that maintains cancer cell homeostasis. Long non-coding (Lnc) RNAs control cell proliferation and play an important role in the occurrence and progression of colorectal cancer (CRC). ZCCHC4 is a newly discovered m6A methyltransferase whose role and mechanism in tumors have not yet been elucidated.

**Methods:**

The EpiQuik m6A RNA methylation kit was used to detect the level of total RNA m6A in six types of digestive tract tumors. The Kaplan-Meier method and receiver operating characteristic curve were used to evaluate the prognostic and diagnostic value of the newly discovered m6A methyltransferase, ZCCHC4, in CRC. The effects on CRC growth in vitro and in vivo were studied using gain- and loss-of-function experiments. The epigenetic mechanisms underlying ZCCHC4 upregulation in CRC were studied using RIP, MeRIP-seq, RNA pull-down, and animal experiments.

**Results:**

We reported that the ZCCHC4-LncRNAGHRLOS-KDM5D axis regulates the growth of CRC *in vitro and in vivo*. We found that ZCCHC4 was upregulated in primary CRC samples and could predict adverse clinical outcomes in patients with CRC. Mechanistically, ZCCHC4 downregulated LncRNAGHRLOS to promote CRC tumorigenesis. As a downstream molecule of LncRNAGHRLOS, KDM5D directly controls CRC cell proliferation, migration, and invasion.

**Conclusion:**

This study suggests that the ZCCHC4 axis contributes to the tumorigenesis and progression of CRC and that ZCCHC4 may be a potential biomarker for this malignancy.

## Background

Colorectal cancer (CRC) is the second most common malignant tumor of the digestive system worldwide, with high incidence and mortality rates. In China, approximately 376,000 new cases and 191,000 deaths occur annually, and the incidence and mortality rates keep rising [1, 2]. However, most patients show no apparent clinical symptoms in the early stages of the disease due to the lack of early detection indicators or characteristic molecular markers. Thus, most patients are usually diagnosed in the late stages and lose the opportunity for surgery or other treatments [3, 4].

In the 1980s, scientists found that when the nuclear DNA sequence did not change, gene function could undergo reversible and heritable changes [5, 6]. This field is referred to as epigenetics. DNA methylation is a well-known method for epigenetic modification. Similarly, there are many chemical modifications on eukaryotic RNA, including N6 methyladenine (m6A), N7 methylguanine (m7G), and N1 methyladenine (m1A), among which m6A is the most abundant chemical modification. Regarding m6A, three important components have been identified: methyltransferase, demethylase, and m6A binding protein. Methyltransferase is figuratively called “Writer,” and demethylase is called “Eraser.” The m6A binding protein recognizes m6A and is called the “Reader.” m6A is a dynamically regulated reversible chemical modification. Some studies have shown that once primary stimuli such as hypoxia occur, it leads to the uncontrolled expression of these genes, leading to angiogenesis and tumor growth progress [7–10].

In 2012, the m6A modification map of the entire human transcriptome was first revealed using methylated RNA immunoprecipitation combined with high-throughput sequencing (MeRIP-seq) technology [11, 12]. Since then, RNA m6A modification has attracted increasing attention from scientists. In addition to mRNA, m6A modification occurs in various non-coding RNA (ncRNAs), such as tRNA, rRNA, snRNA, snoRNA, and long non-coding RNA (lncRNA). For example, m6A modification occurs on LncRNA-MALAT1, which is closely related to dynamic atherosclerosis and is considered to be closely related to changes in the stem ring structure [13–18].

The role of m6A in various diseases, especially cancer, has attracted increasing attention. Many studies have confirmed that changes in the m6A modification intensity widely affect the progression of various diseases, and research in colorectal cancer is becoming increasingly in-depth [19–23]. The comprehensive extent of m6A modification is under dynamic regulation, with both its “writer” and “eraser” contributing to the regulatory process. Although the roles of methyltransferases in CRC have been widely reported, the newly discovered involvement of methyltransferases in CRC progression will continue to enhance our understanding of m6A. In this study, we identified the role of ZCCHC4, a key methyltransferase, in promoting the proliferation, migration, and metastasis of CRC cells. We conducted additional screening of essential downstream molecules and unveiled the mechanism by which ZCCHC4 modulates the behavior of CRC cells.

## Materials and methods

### CRC samples from patients and cell lines

Tumors and adjacent tissues of hepatocellular carcinoma (HCC), esophageal cancer (EC), gastric cancer (GC), gallbladder cancer, CRC, and pancreatic cancer (PC) were procured from patients at the Department of General Surgery, The First Affiliated Hospital of Anhui Medical University. The samples were promptly stored in liquid nitrogen for preservation, and informed consent was obtained from each participant. Human CRC cell lines were acquired from the National Collection of Authenticated Cell Cultures and cultured in Dulbecco’s Modified Eagle Medium (DMEM) containing 10% Fetal Bovine Serum (FBS; Gibco, Waltham, MA, USA) and 1% penicillin-streptomycin (Beyotime, Shanghai, China).

### Constructs and transfections

Genepharm (Shanghai, China) designed and synthesized short hairpin RNA (shRNA) interfering with the expression of ZCCHC4, LncGHRLOS, and KDM5D. Overexpression of ZCCHC4, lncGHRLOS, and KDM5D was performed using expression plasmids, and empty vectors were used as negative controls. The cells were transfected with the virus in the presence of a polycoagulant. After 48 h, puromycin was added to the medium to identify stable clones. The efficacy of overexpression was assessed using qRT-PCR and western blot analyses. The transfection efficiency was evaluated by qRT-PCR and western blotting after 48 h.

### The m6A RNA methylation assay

Total RNA was extracted according to the manufacturer’s instructions. The m6A RNA methylation quantitative kit (Abcam, Cambridge, UK) was used to determine the RNA m6A levels in total RNA. First, the RNA packets were incubated in wells for 90 min at 37 °C. Capture antibody, detection antibody, and booster solution were added. Finally, a color-developing solution was added to detect the signal and measure absorbance. The calorimetric m6A levels were quantified and calculated using a standard curve.

### Cell cycle distribution

Cell cycle analysis was performed using the Muse Cell Cycle Kit and Muse Cell Analyzer (Millipore, Bedford, MA, USA). HCT-15 cells (5 × 10^5^ cells/well) were treated with betulin (0–8 µM) for 24 h in 6-well plates. Propidium iodide (PI)-positive cells were stained and analyzed according to the manufacturer’s instructions.

### Enzyme-linked immunosorbent assay (ELISA)

All blood samples were collected using anticoagulant tubes for further experiments. Each sample contained 5 ml of blood which was centrifuged at 3500 rpm for 10 min. The supernatant plasma was manually separated and stored at -80 ℃ for batch analysis. The concentration of KDM5D was determined at the Central Laboratory of the First Affiliated Hospital of Anhui Medical University. All samples were collected in batches using commercially available kits in accordance with the manufacturer’s instructions.

### Immunohistochemistry (IHC)

Human CRC sections were obtained from the Department of Pathology of the Second Affiliated Hospital of Anhui Medical University. After dewaxing, the sections were repaired in citrate buffer at 95-100°C for 10 min, and incubated with antibodies against ZCCHC4(1:500) and KDM5D (1:400). A vertical microscope (Nikon, Tokyo, Japan) was used for image acquisition. The integrated optical density (IOD) of the stained slices was evaluated using the Image-Pro Plus software (Media Cybernetics, Rockville, MD, USA).

### Western blot

PRO-PREP™ Protein Extraction Solution (iNtRon Biotech, Seoul, Korea) was used to extract total protein from both cells and tissues. The lysates were subsequently combined with a 5 × sample buffer volume and separated by gel electrophoresis to isolate total proteins. The specific target proteins were detected using antibodies obtained from Abcam. Secondary antibodies were used to bind to specific primary antibodies, and the FluorChem M System (ProteinSimple, San Jose, CA, USA) was used for visualization.

### Animal models

Five-week-old male BALB/c nude mice aged 5 weeks were obtained from SPF Beijing Biotechnology Co. Ltd. (Beijing, China). After random assignment, HCT-116 cells suspended in 100 µl phosphate-buffered saline (PBS) were injected subcutaneously into nude mice. All nude mice were fed in an SPF environment and given sufficient water and food, and the tumor growth rate was detected. After 21 days, eight nude mice in the ZCCHC4 group were monitored for tumor progression using molecular imaging software (the other two were not monitored using live imaging as controls). Luciferase signal strength was maintained at the same scale. After in vivo imaging, the mice were killed, the tumors were photographed, weighed, and the tumor tissues were collected for immunohistochemical analysis. All procedures are carried out in accordance with the laboratory animal management code. LncGHRLOS and KDM5D group directly killed the mice, and the tumors were photographed and weighed and stained by IHC. In addition, 5-week nude mice in each group were used to monitor the survival conditions with tumors, and the death time of mice was recorded and the survival curve was drawn.

### RNA extraction and quantitative real-time PCR (qRT-PCR)

The FastPure Cell/Tissue Total RNA Isolation Kit V2 (Vazyme Biotech Co., Ltd. Nanjing, China) was used, and reverse transcription and qRT-PCR for analyzing mRNA expression were conducted using the PrimeScriptTM RT reagent kit (TAKARA, Beijing, China) and TB GREEN SuperMix (TAKARA). The experiment was performed in triplicate.

### RNA-seq, methylated RNA immunoprecipitation sequencing (MeRIP-seq)

Briefly, RNA was extracted and purified to deplete ribosomal RNA and prevent DNA contamination. After fragmentation and denaturation, RNA was sheared into approximately 100-nt fragments and then incubated with an anti-m6A antibody (Abcam) together with protein A/G magnetic beads (Thermo Fisher Scientific, Waltham, MA, USA) in an immunoprecipitation buffer at 4℃ overnight. An RNase inhibitor was added. The antibody combined with methylated RNA was eluted with m6A and purified for MeRIP sequencing using BioAcme (Wuhan, China).

### RNA immunoprecipitation (RIP)

A Magna RIP RNA-binding protein immunoprecipitation kit (Millipore) was used. Briefly, protein A/G magnetic beads conjugated with rabbit immunoglobulin G (17–700, Millipore), ZCCHC4 (Proteintech, USA), or LncGHRLOS (Proteintech, Rosemont, IL, USA) antibodies were incubated with cell lysates supplemented with RNase inhibitor at 4 °C overnight. After washing 6 times, RNA–protein complexes were added to proteinase K buffer. Finally, the RNA was extracted using phenol–chloroform method. qPCR was performed to determine the relative interactions.

### Migration and invasion assays

For the transwell assay, chambers were prepared in a 24-well culture table with 800 ul DMEM containing 10% FBS at the bottom. Then, 5 × 10^4^ cells in 200 ul serum-free medium were added into the upper layer. After 24 h, the migrated cells in the lower layer were fixed, stained with crystal violet, and imaged. Matrigel (Corning, NY, USA) was added to the upper layer for the invasion assay.

### Wound healing assay

CRC cells were plated in 24-well plates and incubated for 24–48 h until 100% growth was achieved. Then, 200 ul micropipette tips were used to generate wounds. The cells were washed twice with 500 ul PBS. Next, 500 uL serum-free medium was added to the well. Images were captured for 24 h and analyzed using ImageJ software (National Institute of Health, Bethesda, MD, USA).

### RNA stability assay

The CRC cells were grown in 12-well plates. Actinomycin D (5 µg/mL, Cell Signaling Technology, Danvers, MA, USA) was used in this experiment. The cells were collected at constant time points for RNA extraction. The remaining LncRNAs were analyzed by qRT-PCR. The half-life of the mRNA was calculated using linear regression analysis and GAPDH was used for normalization.

### RNA pull-down and mass spectrometry analysis

Biotin-labeled LncGHRLOS ssRNA probes were synthesized in vitro by Sangon Biotin (Shanghai, China), while the LncGHRLOS-CDS, CDS-mut1, and CDS-mut2 mRNA were first transcribed using the MEGAscript T7 Transcription Kit (Thermo Fisher Scientific), and then labelled with biotin using Pierce RNA 3′ End Desthiobiotinylation Kit (Thermo Fisher Scientific). Next, 20 pmol of biotinylated RNA and cell lysate were mixed with streptavidin agarose beads (Thermo Fisher Scientific) at 4 ℃ overnight. After six washes, streptavidin beads were collected for western blotting and mass spectrometry.

### Statistical analysis

Statistical analyses were performed using GraphPad Prism 9 software (GraphPad Software, San Diego, CA, USA). Two-tailed unpaired Student’s t-test or one-way analysis of variance were used. Spearman’s correlation analysis evaluated the relationship between the expression of ZCCHC4, lncGHRLOS, and KDM5D. Survival curves of patients with CRC were analyzed using Kaplan–Meier analysis. Receiver operating characteristic (ROC) curves were generated using GraphPad Prism 9. Each experiment had a minimum of three replications.

## Results

### ZCCHC4 is upregulated in CRC tissues and predicted poor prognosis in CRC

**Fig. 1:**
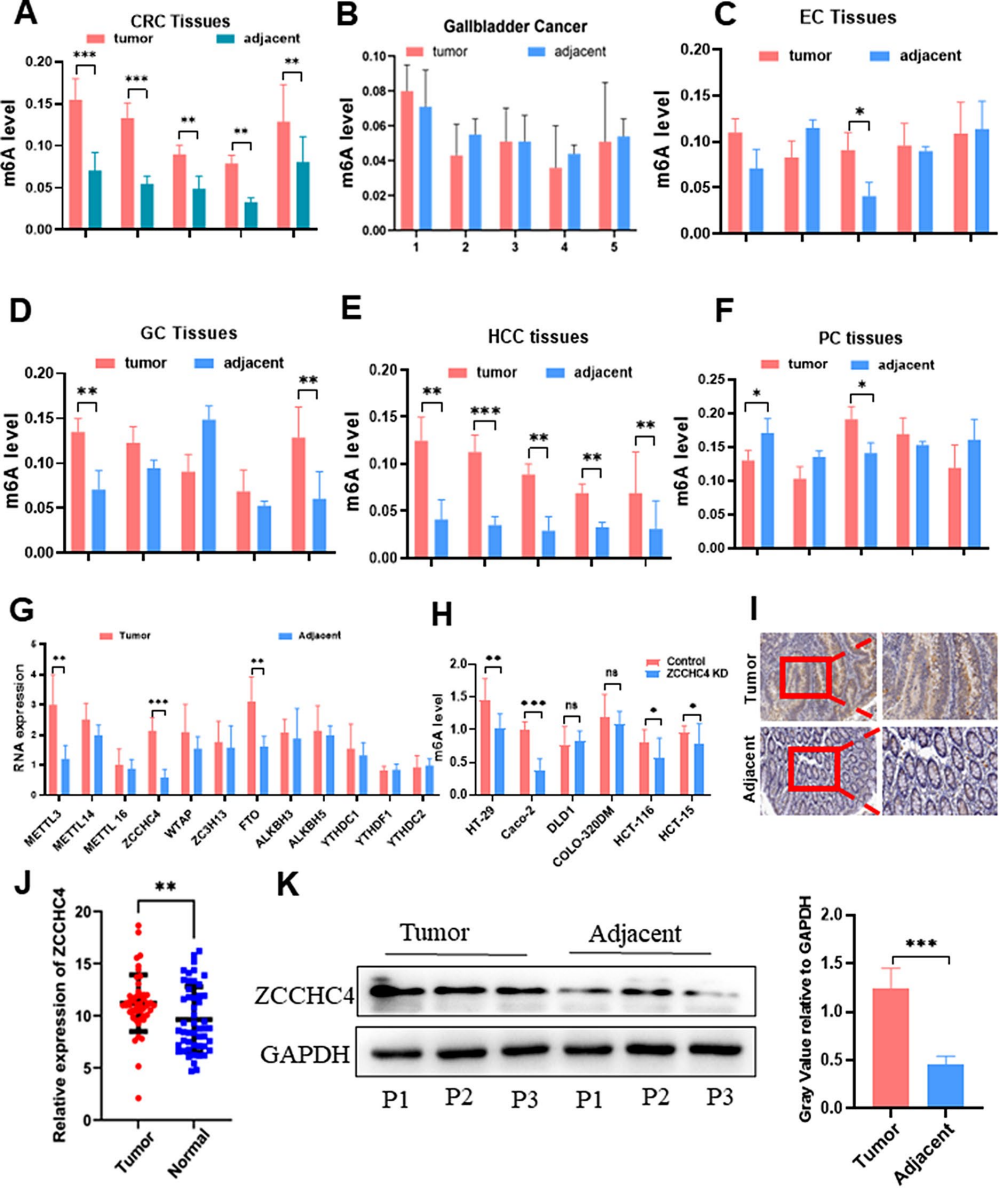
An association between m6A and digestive tract tumors was revealed. (**A-F**) from M6A mRNA levels in 5 pairs of clinical tumor tissues (colorectal, esophageal, gastric, pancreatic, hepatocellular, and gallbladder cancers) with adjacent normal samples. (**G**) mRNA levels of m6A components were detected in 5 pairs of CRC tissues and adjacent tissues. (**H**) After ZCCHC4 was knocked down, the m6A content in each CRC cell line was detected to decrease accordingly. (**I**) The expression of ZCCHC4 was verified by immunohistochemical staining in CRC and adjacent tissues. (**J**) ZCCHC4 mRNA level were detected by qRT-PCR in 50 consecutive CRC cohorts. (**K**) The protein expression of ZCCHC4 in 3 pairs of CRC and adjacent tissues was verified by western blot

To determine the extent of m6A expression across different digestive tract tumors, five pairs of clinical tumor tissues with adjacent tissue samples were randomly selected to assess the overall levels of m6A modification (Fig. 1A-F). Significantly higher levels of m6A were observed in HCC and CRC tissues than in the adjacent normal tissues. Considering the extensive research on m6A in HCC, we selected CRC for this study. Similarly, we detected some key m6A components in five CRC specimens. Finally, we confirmed significant differences in METTL3, FTO, and ZCCHC4 expression between cancerous and adjacent tissues (Fig. 1G). After the ZCCHC4 knockout, a decline in m6A expression was observed in most CRC cell lines (Fig. 1H). The role and mechanism of ZCCHC4 in CRC have become a potential research topic.

To investigate whether ZCCHC4 is abnormally expressed in CRC, IHC was used to detect the expression level of ZCCHC4 in 243 patients with CRC, and qPCR analysis was used to explore the expression of ZCCHC4 mRNA in 30 CRC tissue samples. IHC results showed that ZCCHC4 was highly expressed in the tumor tissues of 175 patients, while patients with low expression accounted for only 26.47% (Fig. 1I). The mRNA levels of ZCCHC4 were higher in most CRC tissues (Fig. 1J). Similar results were obtained after western blot analysis of the three pairs of CRC and adjacent cancer tissues (Fig. 1K).

**Fig. 2:**
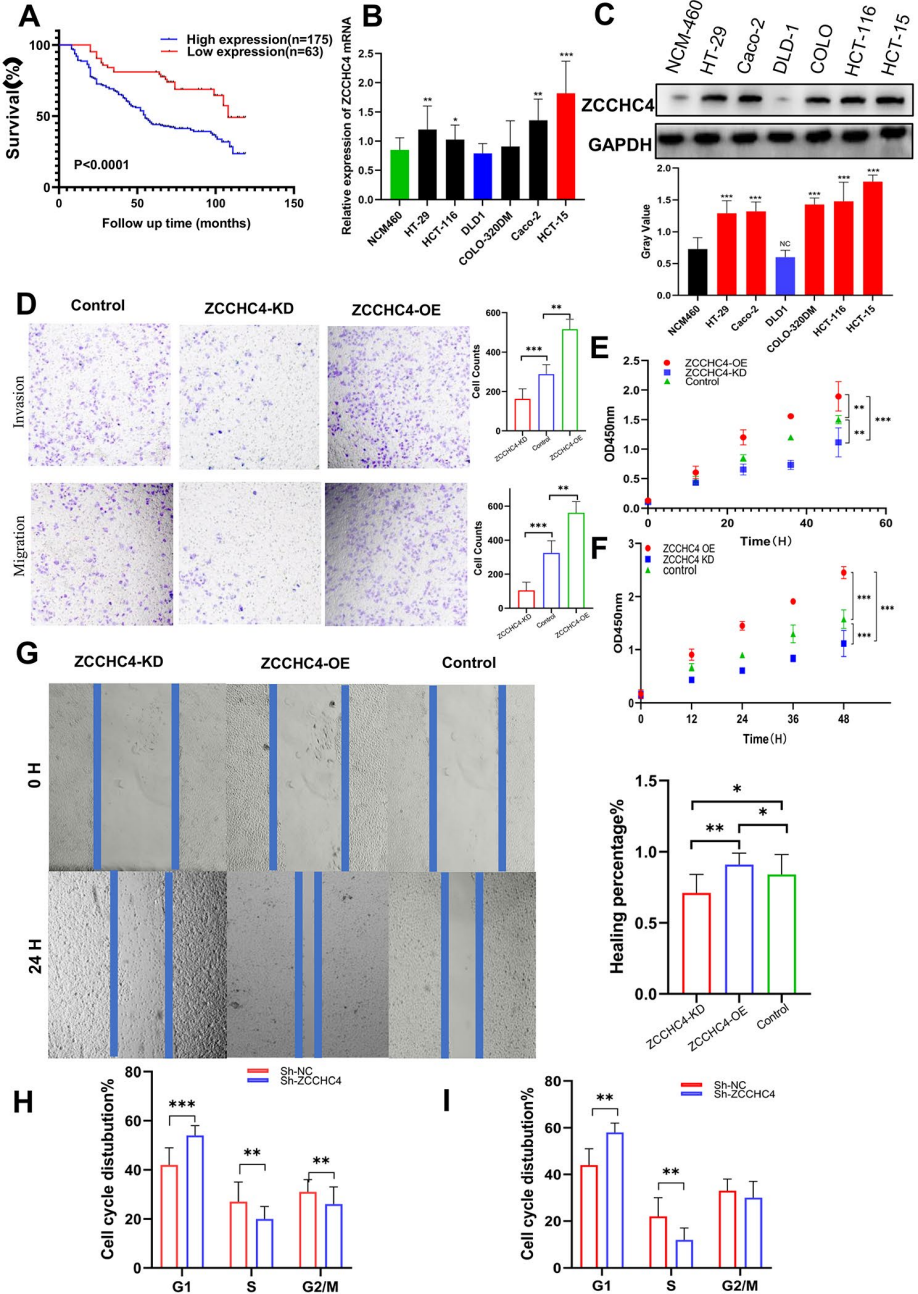
ZCCHC4 is associated with CRC prognosis and the biological behavior of CRC cells in vitro. (**A**) The relationship between overall survival and ZCCHC4 expression was analyzed in 238 consecutive cases of CRC. (**B**) The bar graph describes the ZCCHC4 mRNA levels of different CRC cell lines compared with normal intestinal mucosal epithelial cell lines (NCM-460). (**C**) Expression levels of ZCCHC4 protein in different CRC cell lines Transwell tests (**D**), CCK-8 (**E, F**) and wound healing tests (**G**) demonstrated that down-regulation of ZCCHC4 inhibited the proliferation, migration and invasion of CRC cells, while up-regulation of ZCCHC4 induced the opposite effect. Flow cytometry down-regulated ZCCHC4 in HCT-116 (**H**) and HCT-15 (**I**) cells to block the cell cycle in G1 phase

Between January 2015 and July 2017, postoperative specimens and clinical and pathological information related to patients with CRC were fully preserved. The relationship between ZCCHC4 expression and clinicopathological variables was also explored, and its expression was found to be closely related to the TNM stage and differentiation degree (Table 1). Next, we studied the relationship between ZCCHC4 levels and the prognosis of patients with CRC. The results of Cox regression model analysis showed that patients with higher levels of ZCCHC4 had a shorter overall survival (OS) compared with those with low levels of ZCCHC4 (Table 2). All the patients had complete follow-up data. After 60–77 months of follow-up, the results showed that the prognosis of patients with CRC with high ZCCHC4 expression was significantly worse than that of the low expression group (*P* < 0.0001, Fig. 2A). These results indicated that high ZCCHC4 expression was associated with poor OS.

**Table 1:** Associations of ZCCHC4 expression with clinical parameters in CRC

Varibles		ZCCHC4 expression		*P* value
		Low(*n* = 68)	High(*n* = 175)	
Gender	Female	33	69	0.104
	Male	35	106	
Age	>55	49	112	0.076
	≤ 55	19	63	
Tumor size	>3.5 cm	44	90	0.081
	≤ 3.5 cm	24	85	
Vascular invasion	Positive	15	42	0.102
	Negative	53	133	
Nerve invasion	Positive	13	37	0.113
	Negative	55	138	
Differentiation degree	High/median	40	71	**0.027**
	Low	28	104	
Depth of infiltration	T1/T2	29	80	0.097
	T3/T4	39	95	
Positive lymph nodes	≥ 3	20	84	0.0877
	<3	40	99	
TNM stage	I/II	41	66	**0.008**
	III/IV	27	109	

**Table 2:** Univariate and multivariate analyses of survival in patients with CRC (Cox proportional hazards regression model)

Variables	Univariate analysis			Multivariate analysis		
	HR	95%CI	*P*	HR	95%CI	*P*
**Age**	1.315	0.906–1.909	0.149			
>55 VS ≤ 55						
**Gender**	0.885	0.670–1.412	0.973			
Female VS male						
**Tumor site**	1.191	0.786–1.806	0.409			
Colon VS Rectum						
**Tumor size**	1.419	0.922–2.184	0.111			
>3.5 cm VS ≤ 3.5 cm						
**Differentiation degree**	4.153	2.501–6.896	0.000	2.406	1.635–3.542	0.001
Poor VS High/median						
**Vascular invasion**	1.333	0.872–1.811	0.203			
Positive VS negative						
**Nerve invasion**	1.020	0.777–1.295	0.265			
Positive VS negative						
**TNM stage**	1.972	1.300–2.991	0.001	2.442	1.291–4.620	0.006
I/II VS III/IV						
**ZCCHC4 expression**	2.503	1.71–3.663	0.000	2.352	1.467–3.772	0.000
Decreased VS Non-decreased						

### ZCCHC4 is associated with the biological behavior of CRC cells in vitro and in vivo

**Fig. 3:**
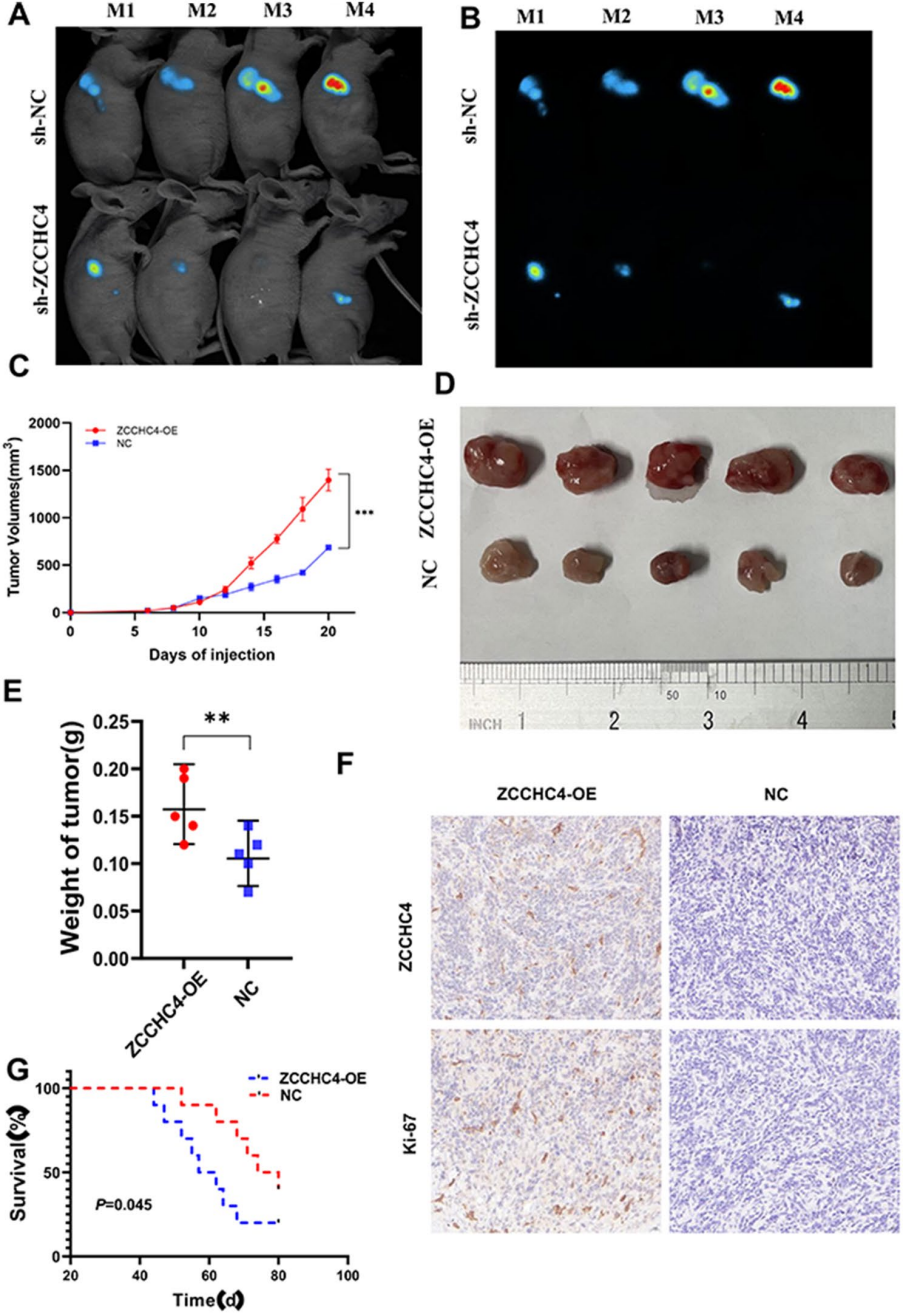
ZCCHC4 promotes tumor growth in vivo. NC or ZCCHC4-OE HCT-116 cells were implanted subcutaneously in nude mice, and tumor nodules were formed subcutaneously in both groups. (**A-B**) In vivo imaging showing the size and location of the tumors in the two groups of mice. (**C**) subcutaneous tumor volume growth curves in the two groups of mice. (**D**) Size of the tumor mass. (**E**) comparison of the weight of the tumors in the mice after death. (**F**) Comparison of survival time between the two groups of mice. (**G**) detection of ZCCHC4 and Ki67 indexes in tumor samples of the two groups by IHC

To determine the potential role of ZCCHC4 in CRC progression, we first assessed the expression of ZCCHC4 among CRC cell lines. We detected ZCCHC4 levels in several CRC cell lines and found that both the mRNA and protein levels of ZCCHC4 were elevated in most CRC cell lines when compared with the normal colorectal epithelial cell line NCM460 (Fig. 2B-C). Subsequently, stable overexpression and knockdown of ZCCHC4 were established in HCT-15 cells. Transwell cell experiments were conducted to investigate the effect of ZCCHC4 on the invasive and migratory abilities of CRC cells (Fig. 2D). The results showed that in the HCT-15 cell line, downregulation of ZCCHC4 expression significantly reduced the invasion and migration abilities of CRC cells compared to the control group. As expected, the CCK-8 assay revealed that the downregulation of ZCCHC4 markedly inhibited the proliferation of HCT-15 and HCT-116 cells (Fig. 2E-F). These results were confirmed by a wound healing assay experiment (Fig. 2G). By examining the cell cycle, it was observed that downregulation of ZCCHC4 expression led to a higher proportion of CRC cells in the G1 phase in both HCT-116 and HCT-15 cell lines (Fig. 2H-I). Given that ZCCHC4 is crucial for maintaining CRC cell proliferation, we validated the in vivo role of ZCCHC4 in CRC tumorigenesis using a nude mouse xenograft model. HCT-116 cells with stable and low ZCCHC4 expression mediated by lentiviruses were subcutaneously inoculated into nude mice to study the tumorigenicity of ZCCHC4 in CRC cells. It is worth noting that the high expression group of ZCCHC4 showed increased growth ability of CRC cells in nude mice, resulting in more volume, as confirmed by in vivo imaging (Fig. 3A-D), increased tumor weight (Fig. 3E) and poor prognosis (Fig. 3F). IHC results showed a decrease in the molecular marker Ki-67 for cell proliferation in ZCCHC4 deficient tumors compared to overexpression group, suggesting that ZCCHC4 may be a driving factor for CRC tumor development (Fig. 3G).

### LncGHRLOS was identified as a downstream target of ZCCHC4

**Fig. 4:**
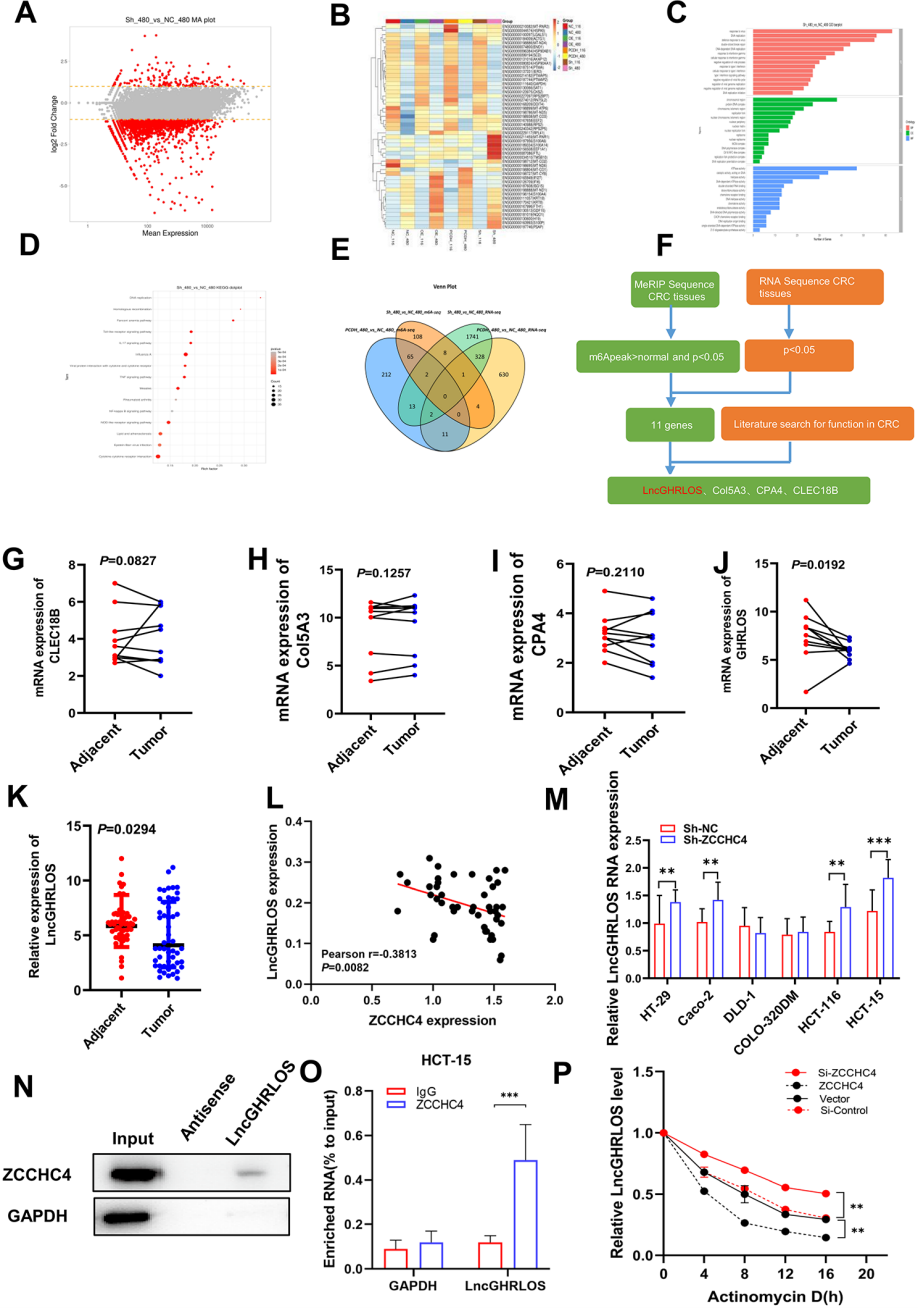
LncGHRLOS was identified as a downstream target of ZCCHC4. (**A**) Volcano map can be used to visualize the overall distribution of differentially expressed genes. Two levels of difference multiple (log2(Fold change)) and significance level were evaluated. (**B**) Gene expression cluster analysis is used to judge the clustering pattern of gene expression in different treatments. (**C**) The protein sequences of all genes and found differentially expressed genes (gene sequences) were compared with Uniport database protein sequences, and then the comparison results were annotated by GO function according to the known protein GO annotation in Uniport database, and the pathway significance was tested using hypergeometric distribution. The GO Terms screening condition for significant enrichment was the *p* value of hypergeometric distribution test less than 0.05. (**D**) The identified differentially expressed genes were enriched by KEGG metabolic pathway using the clusterpro digestion package in R language. (**F**) Filtering process of target Genes among MeRIP-seq, RNA-Seq and literature search. (**G-J**) CLEC18B, Col5A3, CPA4 and LncGHRLOS mRNA levels were detected in 10 CRC and adjacent tissue samples (**K**) LncGHRLOS mRNA levels in 50 CRC cancer tissues and adjacent tissues were detected by qRT-PCR. (**L**) ZCCHC4 and LncGHRLOS were verified in 50 continuous CRC cases correlation between endogenous LncGHRLOS mRNA levels (**M**) Detection of LncGHRLOS mRNA levels after endogenous LncGHRLOS using NC or ZCCHC4 by lentivirus in different CRC cell lines. (**N**) RNA pulldown of endogenous LncGHRLOS using NC or ZCCHC4 probe. (**O**) RIP-qPCR assay of ZCCHC4 enrichment by LncGHRLOS in HCT-15 cell lines. (**P**) RNA stability of LncGHRLOS mRNA after treatment with ZCCHC4 overexpression and knockdown

To explore the potential targets of ZCCHC4 in CRC, we conducted RNA analysis of control and CRC cell lines expressing ZCCHC4. RNA-seq results showed that most transcripts were downregulated after ZCCHC4 overexpression. A volcano map was used to visualize the overall distribution of differentially expressed genes. Two levels of difference, multiple (log2(fold change)) and significance, were evaluated (Fig. 4A). Gene expression cluster analysis was used to determine the clustering patterns of gene expression in different treatments (Fig. 4B). The protein sequences of all genes and identified differentially expressed genes (gene sequences) were compared with protein sequences from the UniProt database, and the comparison results were annotated by GO function according to the known protein GO annotation in the UniProt database and the pathway significance was tested using the hypergeometric distribution. The GO term screening condition for significant enrichment was a *p*-value of hypergeometric distribution test less than 0.05(Fig. 4C). The identified differentially expressed genes were enriched in the KEGG metabolic pathway using the “clusterpro digestion” package in R (Fig. 4D). In order to identify the main target genes that play a critical role downstream of ZCCHC4, based on MeRIP-seq results and combined with the genes that significantly changed after overexpression of ZCCHC4 in RNA seq results, we identified 11 common genes (Fig. 4E). After a literature search, we selected CPA4, Col5A3, lncGHRLOS, and CLEC18B as the potential downstream targets of ZCCHC4(Fig. 4F). After detecting the mRNA expression of these molecules in 10 pairs of CRC tissues and adjacent normal tissues, we found that only lncGHRLOS differed between the two groups, and we used lncGHRLOS as our candidate molecule for further verification (Fig. 4G-J). We first described the correlation between lncGHRLOS and ZCCHC4 using PCR results for the above 50 samples, and the results showed that the two presented a negative correlation (Fig. 4K-L). Moreover, after knocking down the expression of ZCCHC4, we detected significantly increased lncGHRLOS mRNA levels in the CRC cell lines (Fig. 4M). RNA pull-down and RIP experiments also showed binding between ZCCHC4 and lncGHRLOS transcripts (Fig. 4N-O). Notably, ZCCHC4 ablation slowed the degradation of lncGHRLOS in CRC cells, whereas ZCCHC4 overexpression accelerated the degradation of lncGHRLOS transcripts (Fig. 4P). Therefore, we speculate that lncGHRLOS may be a downstream effector of ZCCHC4.

### LncGHRLOS inhibited the proliferation, invasion, and migration of CRC in vitro and in vivo

**Fig. 5:**
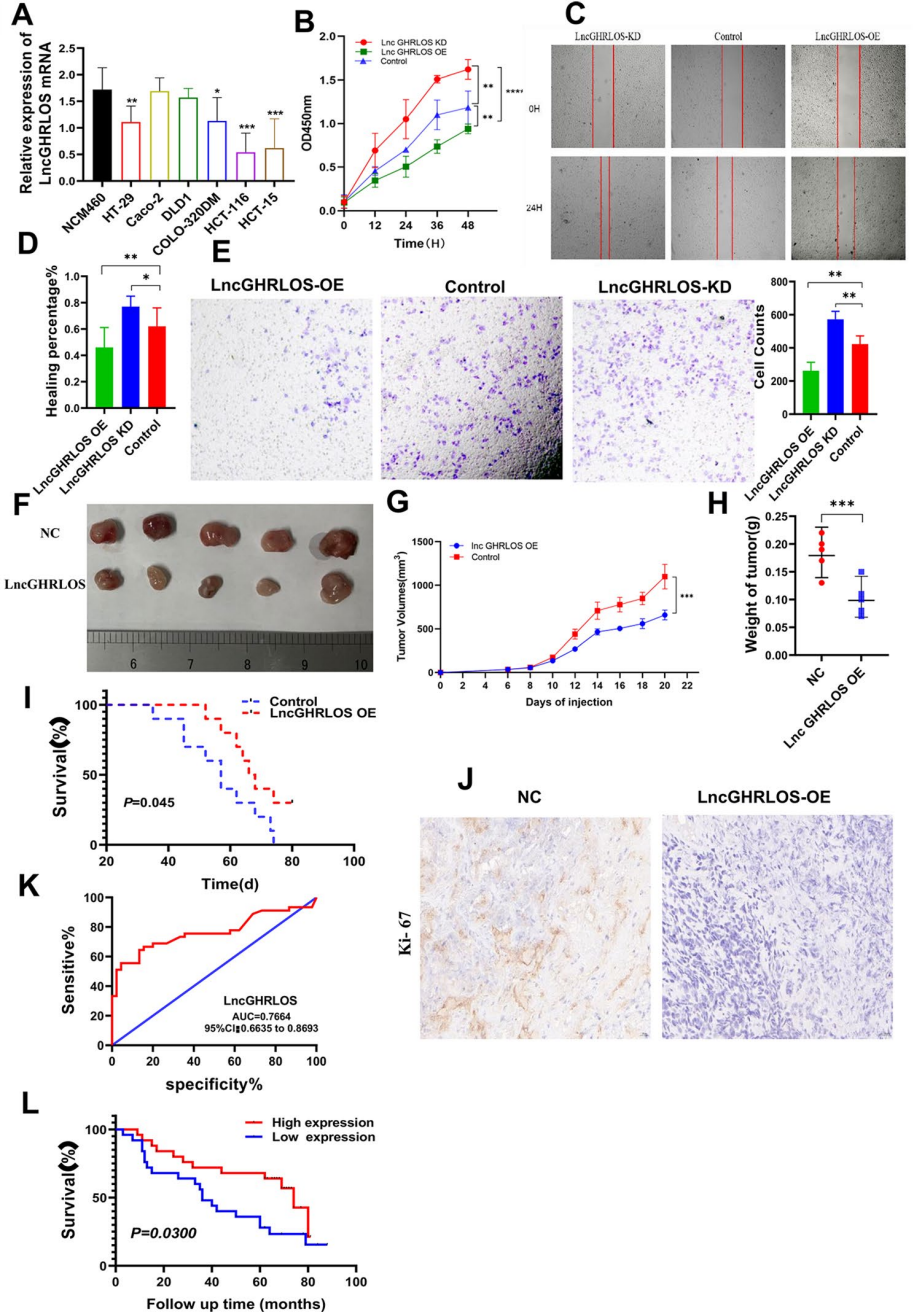
LncGHRLOS inhibits the tumorigenesis of CRC in vitro and in vivo. (**A**) LncGHRLOS mRNA expression was analyzed by qRT-PCR in normal intestinal mucosal epithelial cells and 6 CRC cell lines, and LncGHRLOS was down-regulated in most cell lines. (**B**) The effect of HT-29 cell viability after CCK-8 detection. (**C-D**) Wound healing test after overexpression and LncGHRLOS knockdown, injury measurement after 24 H, HT-29 cell line, original magnification :40×, Scale :100 μm. (**E**) Transwell migration assay was used to detect the effect of LncGHRLOS overexpression and knocked down on cell migration in HT-29 cell line. (**F**) The tumor formation model of nude mice was made using HCT-116 cells. The tumor volume was monitored every 2 days from day 6 to day 20 after cell inoculation, and the curve was drawn. Overexpression of LncGHRLOS resulted in reduced tumor volume (**G**) and weight (**H**). (**I**) Changes in the survival time of mice. After overexpression of LncGHRLOS, the survival time of mice was prolonged. (**J**) The number of Ki-67 positive cells in tumor sections was detected by IHC. There were fewer Ki-67 positive cells in tumors with LncGHRLOS overexpression. (**K**) ROC curve shows that LncGHRLOS has a good diagnostic value for CRC. The area under the curve was 0.7664. (**L**) the survival curve of CRC patients was drawn combined with the follow-up information of patients. CRC patients with high expression of LncGHRLOS had a better prognosis

To verify the unique role of lncGHRLOS in CRC, we first analyzed lncGHRLOS in CRC cell lines (Fig. 5A). shRNA was used to specifically reduce the intracellular RNA levels of lncGHRLOS in HCT-15 cells and activate the expression of lncGHRLOS through a lentivirus-mediated system. The proliferative ability of CRC cells treated with lncGHRLOS was detected using a CCK-8 assay. The CCK-8 activity experiment showed that silencing lncGHRLOS effectively enhanced the proliferative ability of CRC cells, while enhanced expression of lncGHRLOS caused the opposite biological effect (Fig. 5B). A wound healing experiment was performed to determine the migration ability of the cells (Fig. 5C-D). Our results showed that after overexpression of lncGHRLOS, the migratory ability was significantly reduced compared to that of ordinary CRC cells, and there was a significant difference compared to the low expression group of lncGHRLOS. The Transwell assay yielded almost identical results (Fig. 5E).

Subsequently, we inoculated nude mice with CRC cells stably overexpressing lncGHRLOS to study the effect of lncGHRLOS on tumorigenicity. Notably, overexpression of lncGHRLOS reduced the growth of CRC cells in nude mice, resulting in a smaller tumor volume (Fig. 5F-G), reduced tumor weight (Fig. 5H), better prognosis (*P* = 0.045, Fig. 5I). Consistent with these observations, IHC results showed that the molecular marker Ki-67 for cell proliferation was downregulated in lncGHRLOS-overexpression tumors compared to the corresponding control group (Fig. 5J). Moreover, samples of patients with CRC with CRC used for PCR detection and ROC analysis showed that lncGHRLOS had significant differentiation in clinical CRC diagnosis (AUC = 0.7664, Fig. 5K). The survival curve of the patients was drawn based on the follow-up results. The results showed that patients in the high lncGHRLOS expression group had a longer survival period (*P* = 0.0300, Fig. 5L).

### Correlation between ZCCHC4, LncGHRLOS and KDM5D

**Fig. 6:**
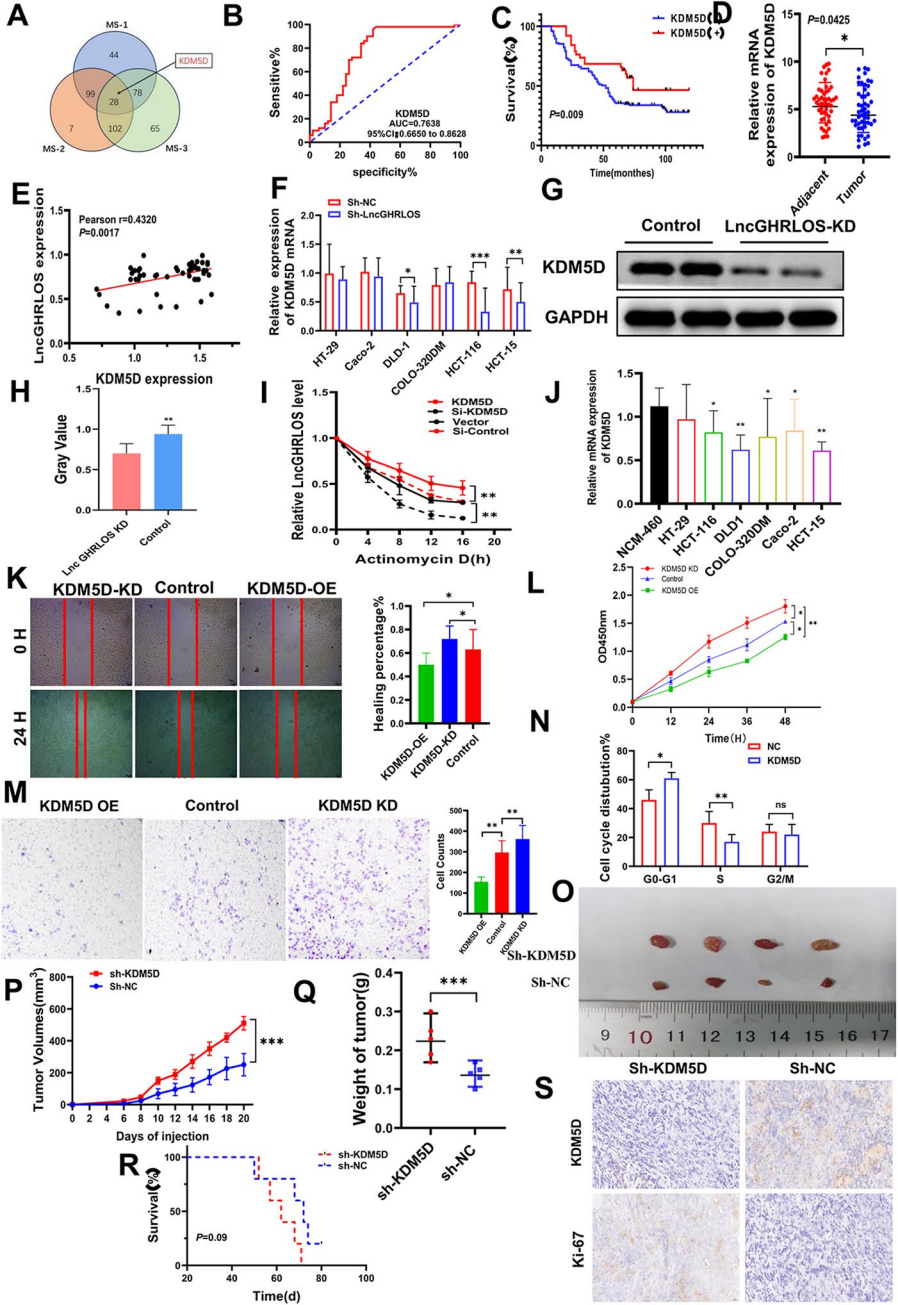
ZCCHC44 and LncGHRLOS co-regulate the expression of KDM5D. (**A**) LncGHRLOS probe mass spectrometry was used for analysis. (**B**) After the expression of KDM5D in tumors was analyzed by qRT-PCR, the survival curve of colorectal cancer patients was drawn combined with the follow-up information of patients. The prognosis of colorectal cancer patients with high expression of KDM5D is better. (**C**) ROC curve showed that KDM5D had good diagnostic value for CRC. The area under the curve is 0.7638. (**D**) KDM5D mRNA levels were detected by PCR in 50 CRC tissues and adjacent normal tissues, and the results showed that the content of KDM5D in adjacent tissues was higher than that in tumor tissues. (**E**) Correlation analysis of KDM5D and LncGHRLOS in tissues of 50 CRC patients. (**F-H**) KDM5D protein was detected by PCR and WB in different colorectal cancer cell lines. (**I**) RNA stability of LncGHRLOS mRNA after treatment with KDM5D overexpression, KDM5D knockout and control cells with actinomycin D (5 µg/mL). (**J**) Significantly reduced expression of KDM5D mRNA was observed in many CRC cell lines. (**K**) Wound healing test after overexpression and KDM5D knockdown, injury measurement after 24 H, original magnification :40×, Scale :100 μm (**L**) The effect of CRC cell viability after CCK-8 detection. (**M**) Transwell migration assay was used to detect the effect of KDM5D overexpression and knocked down on cell migration in CRC cells. (**N**) Flow cytometry up-regulated KDM5D in CRC cells to block the cell cycle in G0/1 phase. (**O**) The tumor formation model of nude mice was made using HCT-116 cells. The tumor volume was monitored every 2 days from day 6 to day 20 after cell inoculation, and the curve was drawn. The tumor volume was monitored every 2 days from day 6 to day 20 after the overexpressed cells were inoculated. Downexpression of KDM5D resulted in increased tumor volume (**P**) and weight (**Q**). (**R**) Changes in the survival time of mice. There was no statistical significance between the two groups. (**S**) The number of Ki-67 positive cells in tumor sections was detected by IHC. There were more Ki-67 positive cells in tumors with KDM5D knockdown

RNA pull-down experiments and mass spectrometry were performed to investigate the potential downstream targets of lncGHRLOS. Mass spectrometry shows that KDM5D may play a role in the m6A modification of LncGHRLOS (Fig. 6A). Using the median of PCR results, we divided patients with high and low expression of KDM5D in clinical specimens into groups and found that KDM5D was poorly expressed in CRC, and its low expression conveyed poor prognosis in patients with CRC (*P* = 0.009, Fig. 6B). ELISA results also showed that KDM5D has the potential to be a marker of CRC (AUC = 0.7638, Fig. 6C). CRC and adjacent tissues were selected for PCR experiments to confirm *KDM5D* mRNA levels (*P* = 0.0425, Fig. 6D). Furthermore, a significant positive correlation between *LncGHRLOS* and *KDM5D* mRNA expression was observed in CRC (*P* = 0.0017, Fig. 6E). Validation experiments in cell lines confirmed that the expression of KDM5D was significantly reduced after the knockdown of LncGHRLOS (Fig. 6F-H). Importantly, we evaluated the stability of lncGHRLOS mRNA after knockdown and overexpression of KDM5D. It was found that the mRNA stability of lncGHRLOS was significantly increased after overexpression of KDM5D under actinomycin D treatment but significantly decreased after knockdown of KDM5D (Fig. 6I). Significantly reduced expression of *KDM5D* mRNA was observed in many CRC cell lines (Fig. 6J). Next, we used a wound healing assay (Fig. 6K), CCK-8 assay (Fig. 6L), transwell assay (Fig. 6M) and flow cytometry (Fig. 6N) to verify the influence of KDM5D knockdown and overexpression on the biological behavior of CRC cells. We found that high expression of KDM5D reduced the proliferation and migration ability of HCT-116 cells. The cell cycle stagnates during the G0/G1 phase. Furthermore, we performed in vivo experiments to clarify the effects of KDM5D on CRC. Tumor formation experiments in nude mice showed that the low expression group of KDM5D showed increased growth ability of CRC cells in nude mice, resulting in more volume (Fig. 6O-P), increased tumor weight (Fig. 6Q) and Ki-67 index (Fig. 6S). However, unlike in previous experiments, there was no significant difference in survival time between the two groups of mice (*P* = 0.09, Fig. 6R). This may be due to a bias caused by the small number of experimental mice.

## Discussion

Cell proliferation, migration, and metastasis are the fundamental differences between tumor and normal cells. Much has been published regarding RNA modifications and disease-related mechanisms in CRC. A deeper understanding of the role of RNA modifications in CRC progression is essential for the search for novel tumor markers and the evolution of therapeutic strategies [24–27]. m6A is the most prevalent type of RNA modification among mRNAs and ncRNAs. The “writer” and “eraser” form a dynamic regulatory process to control the level of m6A in the human body. Several studies suggest that m6A regulators play important roles in various physiological and pathological diseases by regulating the epigenetic transcriptional levels of genes. m6A modification is involved in various human diseases, such as cardiovascular, digestive, reproductive, nervous, and blood system diseases. Although many studies have investigated the role of key m6A components in CRC [28–33], as a newly discovered m6A methylase, the role of ZCCHC4 in disease progression remains largely unknown.

Through the quantitative analysis of m6A in different gastrointestinal tumor samples, we excluded HCC, which has been extensively studied in the past and focused on the role of m6A in CRC. A newly discovered methyltransferase was identified by continuing the analysis of the different components of m6A. In our study, we found that in CRC cell lines, high expression of ZCCHC4 promoted the proliferation, migration, and invasion of CRC cells, suggesting that ZCCHC4 is important for the survival and viability of CRC cells. Subcutaneous tumor-bearing experiments in mice also confirmed that the ZCCHC4 knockout inhibited CRC progression.

ZCCHC4, a member of the zinc finger protein family, is conserved in multicellular biological tissues but does not exist in yeast. ZCCHC4 contains a conserved catalytic domain, DPPF, which is a potential N6 methyladenosine methyltransferase domain [34–36]. In addition, ZCCHC4 contains CCHC Znf and Zf GRF domains [37]. Currently, there are very few reports on ZCCHC4. In January 2019, Chuan et al. found that ZCCHC4 is an m6A-modifying enzyme that mainly methylates 28 S rRNA and interacts with several mRNAs [38]. ZCCHC4 knockout reduced the m6A modification level of 28 S rRNA and the overall translation level and inhibited cell proliferation. Although many rRNA-related m6A modifying enzymes have been identified in bacteria, ZCCHC4 was the first rRNA-modified m6A methyltransferase identified in eukaryotes [39–42]. Currently, little is known about the function of ZCCHC4 in tumors. Our study first detected m6A levels in colorectal cancer tissues and further demonstrated that ZCCHC4 was a key factor in altering m6A levels, which made it possible to observe ZCCHC4, a newly identified methyltransferase from the perspective of m6A for the first time. Although ZCCHC4 can also participate in CRC development as an RBP, its specific mechanism of action has not been elucidated. Instead, m6A-seq continued to be used to search for downstream molecules of ZCCHC4 and finally confirmed that it could bind to lncGHRLOS to affect CRC progression [43–46].

However, we omitted delving into the bioinformatics aspects, primarily because of the contentious nature of ZCCHC4 in CRC. The novelty of ZCCHC4 as an m6A methylase raises concerns about the reliability of the results due to the limited available data. Our study conducted consistent clustering and principal component analysis (PCA) on the GSE 17,536 dataset, encompassing expression profiles and clinical data from 175 patients with CRC with a minimum overall survival of 30 days. The patients were categorized into two distinct groups: colon cancer 1 (*N* = 105) and colon cancer 2 (*N* = 70). Our findings indicated significant associations between RBM 15B, ZCCHC4, KIAA1429, IGF2BP2, and FTO and overall survival (OS) in patients with CRC. Using the median expression value of ZCCHC4 as a cut-off, patients were stratified into high- and low-expression groups. We established KM survival curves to evaluate the prognostic significance of these five genes in 175 patients with CRC from GSE 17,536. Surprisingly, ZCCHC4 did not demonstrate predictive value for OS in patients with CRC. Furthermore, analysis of the other four markers yielded negative results, highlighting the prognostic divergence between patients with negative and positive ZCCHC4 levels. Consequently, a comprehensive bioinformatics analysis is imperative for constructing a polygenic prognostic model.

ZCCHC4 was finally recognized because of its role as a member of the RBP family, which has been shown to play an important role in the development and treatment of cancer [47–50]. However, tumor-promoting RBP and their partners, which may serve as potential cancer therapeutic targets, need to be further identified. The results of this study suggest that ZCCHC4 is abnormally highly expressed in various digestive tract tumor tissues and may be a key regulator of CRC progression, and its knockdown can significantly inhibit tumor progression. Statistical analysis of the clinical features showed that low expression of ZCCHC4 was positively correlated with good prognosis and lymph node metastasis. We speculate that ZCCHC4 may have the opposite effect by regulating key target genes. In terms of mechanism, ZCCHC4 inhibits the apoptosis of CRC cells by interacting with a long-chain non-coding RNA (lncRNA) and promotes the proliferation, migration, and invasion of tumor cells. In conclusion, our study identified ZCCHC4 as a novel predictor of poor prognosis in CRC and a potential treatment target, providing mechanistic insights into its role in cancer progression.

Unlike epigenetic modifications such as DNA methylation and histone acetylation, which regulate gene expression at the transcriptional level, the anatomical mechanism of m6A-modified RNA has greatly improved our understanding of gene regulation at the post-transcriptional level in eukaryotic cells [51, 52]. The dynamic modification of m6A mRNA is necessary to maintain cell growth, metabolism, and differentiation. By regulating the abnormal modification of specific mRNA by m6A, tumor cells maintain self-repair and proliferation under different conditions [53]. In recent years, many studies have focused on the role of m6a methylated mRNA in cancer cells, and some lncRNAs have also been reported [54–58]. However, little is known about the regulatory role of m6A modifications of lncRNAs in CRC. Our study revealed that lncGHRLOS, which is not widely recognized, can combine with ZCCHC4 to control CRC by regulating KDM5D. Few studies have investigated its role in CRC, and no studies have explored its mechanism of action in CRC. Notably, we further confirmed that m6A methyltransferases can regulate the stability of lncRNAs in CRC cells, which expands our understanding of the involvement of lncRNAs in the pathogenesis of CRC.

## Data Availability

Data will be made available on request.

## Declarations

### Animal studies

The animal studies protocol was approved by the animal research ethics committee of Anhui Medical University.
