# Correction: The epigenetic downregulation of LncGHRLOS mediated by RNA m6A methylase ZCCHC4 promotes colorectal cancer tumorigenesis

**DOI:** 10.1186/s13046-024-03005-y

**Published:** 2024-03-12

**Authors:** Ke Chen, Jingcheng Zhang, Lei Meng, Lingshang Kong, Ming Lu, Zhengguang Wang, Wenbin Wang

**Affiliations:** 1 Vascular Surgery Department, Nanjing Drum Tower Hospital Affiliated to Medical School, Nanjing University, Nanjing, China; 2 Department of Surgery, Klinikum Rechts Der Isar, School of Medicine, Technical University of Munich, Munich, Germany; 3 General Surgery Department, The Second Affiliated Hospital of Anhui Medical University, Hefei, China; 4 General Surgery Department, Anhui Provincial Hospital, Hefei, China; 5 General Surgery Department, The First Affiliated Hospital of Anhui Medical University, Hefei, China

**Correction:** **J Exp Clin Cancer Res 43, 44 (2024)**

**https://doi.org/10.1186/s13046-024-02965-5**

Following publication of the original article [1], errors were spotted in some of the entries in Table 1, Table 2 and Fig. 6. Specifically:

**Table:** 

Table number	Row	Column	Incorrect data	Correct data
1	Positive lymph nodes—≥ 3	Low (*n* = 68)	15	20
1	Positive lymph nodes—≥ 3	High (*n* = 175)	101	84
1	Positive lymph nodes—< 3	Low (*n* = 68)	53	40
1	Positive lymph nodes—< 3	High (*n* = 175)	74	99
1	Positive lymph nodes	*P* value	0.121	0.087
2		Variables	LncGHRLOS expression	ZCCHC4 expression

Incorrect Fig. 6

**Fig. 6:**
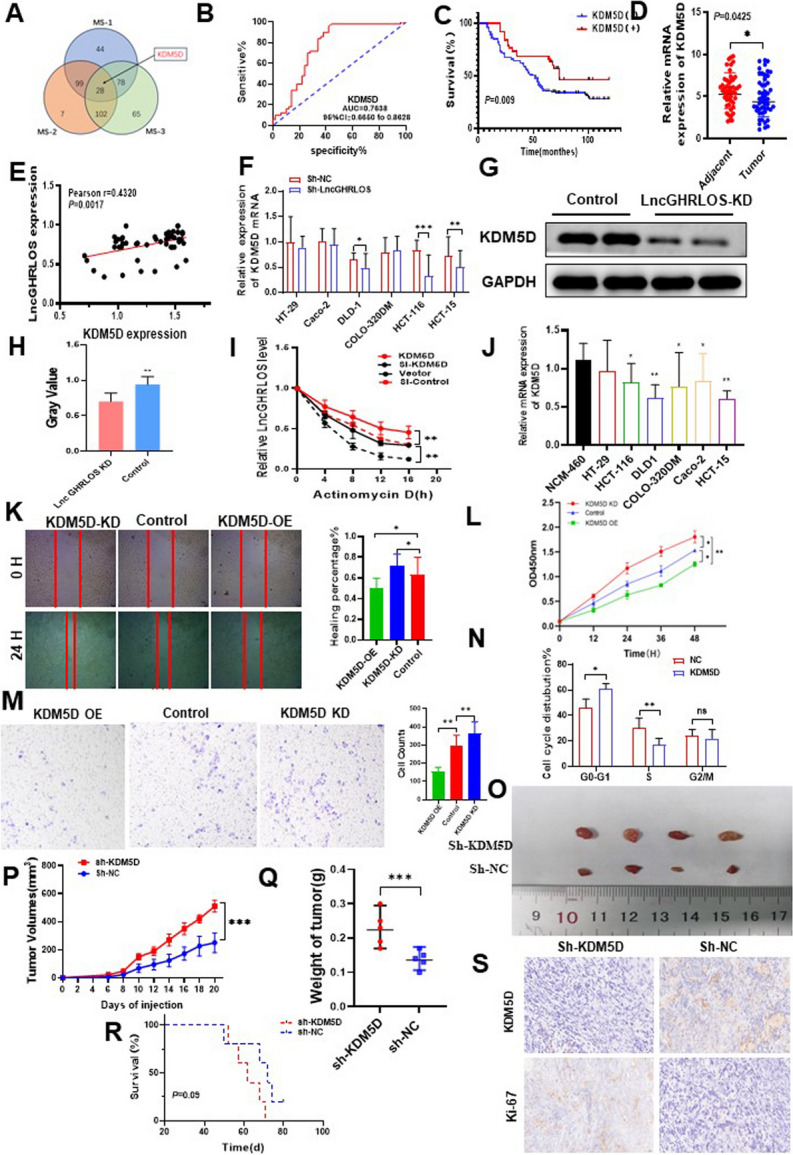
ZCCHC44 and LncGHRLOS co-regulate the expression of KDM5D. **A** LncGHRLOS probe mass spectrometry was used for analysis. **B** After the expression of KDM5D in tumors was analyzed by qRT-PCR, the survival curve of colorectal cancer patients was drawn combined with the follow-up information of patients. The prognosis of colorectal cancer patients with high expression of KDM5D is better. **C** ROC curve showed that KDM5D had good diagnostic value for CRC. The area under the curve is 0.7638. **D** KDM5D mRNA levels were detected by PCR in 50 CRC tissues and adjacent normal tissues, and the results showed that the content of KDM5D in adjacent tissues was higher than that in tumor tissues. **E** Correlation analysis of KDM5D and LncGHRLOS in tissues of 50 CRC patients. **F-H** KDM5D protein was detected by PCR and WB in different colorectal cancer cell lines. **I** RNA stability of LncGHRLOS mRNA after treatment with KDM5D overexpression, KDM5D knockout and control cells with actinomycin D (5 μg/mL). **J** Significantly reduced expression of KDM5D mRNA was observed in many CRC cell lines. **K** Wound healing test after overexpression and KDM5D knockdown, injury measurement after 24 H, original magnification:40 × , Scale:100 μm (**L**) The effect of CRC cell viability after CCK-8 detection. **M** Transwell migration assay was used to detect the effect of KDM5D overexpression and knocked down on cell migration in CRC cells. **N** Flow cytometry up-regulated KDM5D in CRC cells to block the cell cycle in G0/1 phase. **O** The tumor formation model of nude mice was made using HCT-116 cells. The tumor volume was monitored every 2 days from day 6 to day 20 after cell inoculation, and the curve was drawn. The tumor volume was monitored every 2 days from day 6 to day 20 after the overexpressed cells were inoculated. Down expression of KDM5D resulted in increased tumor volume (**P**) and weight (**Q**). **R** Changes in the survival time of mice. There was no statistical significance between the two groups. **S** The number of Ki-67 positive cells in tumor sections was detected by IHC. There were more Ki-67 positive cells in tumors with KDM5D knockdown

Correct Fig. 6

**Fig. 6:**
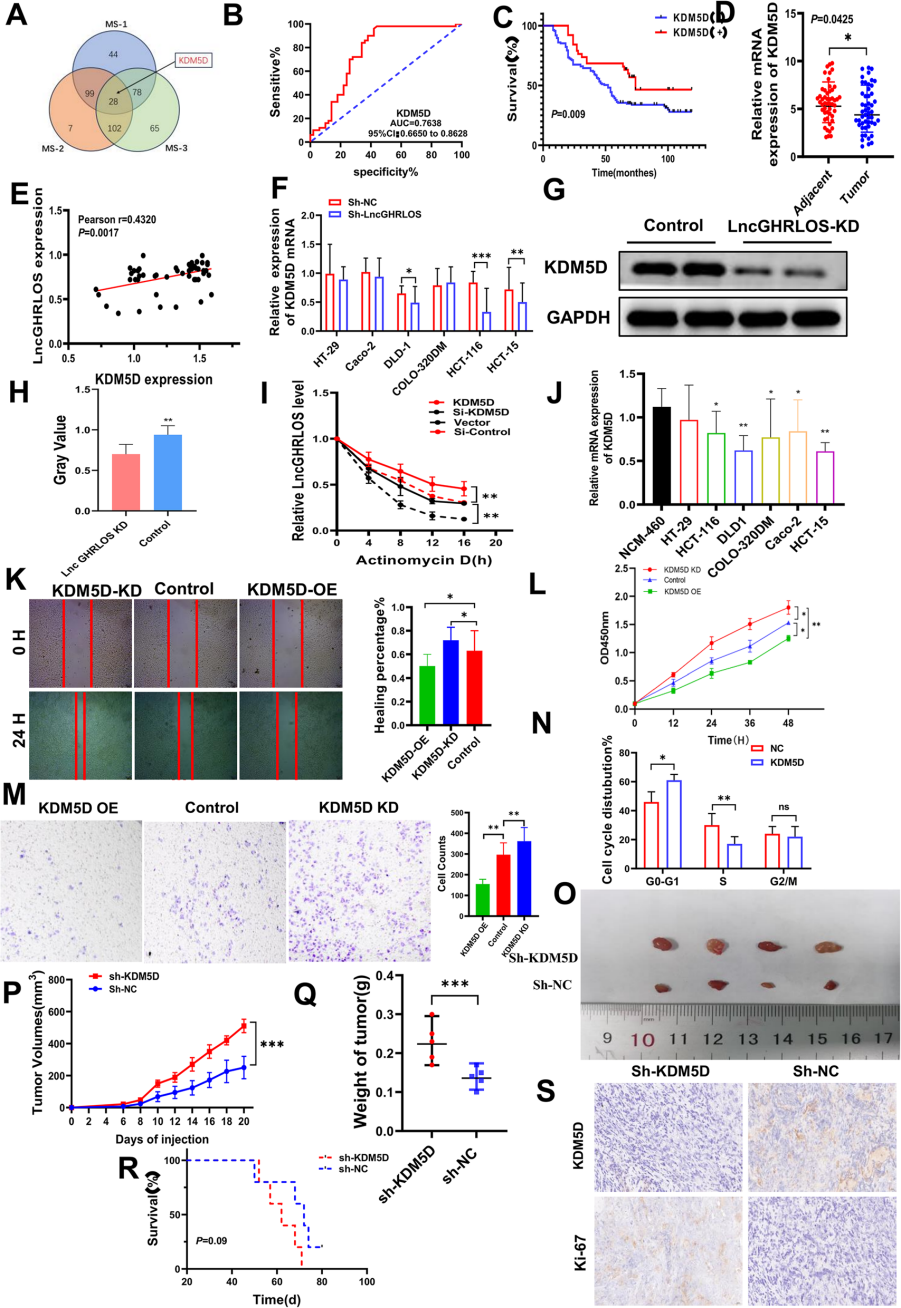
ZCCHC44 and LncGHRLOS co-regulate the expression of KDM5D. **A** LncGHRLOS probe mass spectrometry was used for analysis. **B** After the expression of KDM5D in tumors was analyzed by qRT-PCR, the survival curve of colorectal cancer patients was drawn combined with the follow-up information of patients. The prognosis of colorectal cancer patients with high expression of KDM5D is better. **C** ROC curve showed that KDM5D had good diagnostic value for CRC. The area under the curve is 0.7638. **D** KDM5D mRNA levels were detected by PCR in 50 CRC tissues and adjacent normal tissues, and the results showed that the content of KDM5D in adjacent tissues was higher than that in tumor tissues. **E** Correlation analysis of KDM5D and LncGHRLOS in tissues of 50 CRC patients. **F-H** KDM5D protein was detected by PCR and WB in different colorectal cancer cell lines. **I** RNA stability of LncGHRLOS mRNA after treatment with KDM5D overexpression, KDM5D knockout and control cells with actinomycin D (5 μg/mL). **J** Significantly reduced expression of KDM5D mRNA was observed in many CRC cell lines. **K** Wound healing test after overexpression and KDM5D knockdown, injury measurement after 24 H, original magnification:40 × , Scale:100 μm (**L**) The effect of CRC cell viability after CCK-8 detection. **M** Transwell migration assay was used to detect the effect of KDM5D overexpression and knocked down on cell migration in CRC cells. **N** Flow cytometry up-regulated KDM5D in CRC cells to block the cell cycle in G0/1 phase. **O** The tumor formation model of nude mice was made using HCT-116 cells. The tumor volume was monitored every 2 days from day 6 to day 20 after cell inoculation, and the curve was drawn. The tumor volume was monitored every 2 days from day 6 to day 20 after the overexpressed cells were inoculated. Down expression of KDM5D resulted in increased tumor volume (**P**) and weight (**Q**). **R** Changes in the survival time of mice. There was no statistical significance between the two groups. **S** The number of Ki-67 positive cells in tumor sections was detected by IHC. There were more Ki-67 positive cells in tumors with KDM5D knockdown

The errors were due to author’s miscounting the lymph nodes and miscalculating their *P* value in Table 1. The LncGHRLOS expression in Table 2 was written by mistake and the error in Fig. 6 was due to the assembling mistake in the images.

The original article has been corrected.

## References

[1] Chen K, Zhang J, Meng L (2024). The epigenetic downregulation of LncGHRLOS mediated by RNA m6A methylase ZCCHC4 promotes colorectal cancer tumorigenesis. J Exp Clin Cancer Res.

